# Engineered exosome-mediated delivery of circDIDO1 inhibits gastric cancer progression via regulation of MiR-1307-3p/SOCS2 Axis

**DOI:** 10.1186/s12967-022-03527-z

**Published:** 2022-07-21

**Authors:** Zhen Guo, Yu Zhang, Wenrong Xu, Xu Zhang, Jiajia Jiang

**Affiliations:** 1 Aoyang Institute of Cancer, Affiliated Aoyang Hospital of Jiangsu University, 279 Jingang Road, Suzhou, 215600 Jiangsu China; 2 Jiangsu Key Laboratory of Medical Science and Laboratory Medicine, School of Medicine, Jiangsu University, 301 Xuefu Road, Zhenjiang, 212013 Jiangsu China

**Keywords:** Gastric cancer, CirDIDO1, MiR-1307-3p, SOCS2, Exosomes, Cancer therapy

## Abstract

**Background:**

Our previous study has identified a novel circRNA (circDIDO1) that is down-regulated in gastric cancer (GC) and significantly inhibits GC progression. The purpose of this study is to identify the molecular mechanism for circDIDO1 and to evaluate the therapeutic effect of circDIDO1 in GC.

**Methods:**

By combining bioinformatic analysis with RNA sequencing data, we predicted the potential target of circDIDO1 and further validated the regulatory mechanisms for its tumor suppressor function in GC. RIP assay, luciferase reporter assay and in vitro cell function assays were performed to analyze circDIDO1-regulated downstream target genes. For the therapeutic study, circDIDO1-loaded, RGD-modified exosomes (RGD-Exo-circDIDO1) were constructed and its anti-tumor efficacy and biological safety were evaluated in vitro and in vivo.

**Results:**

CircDIDO1 inhibited GC progression by regulating the expression of the signal transducer inhibitor SOSC2 through sponging miR-1307-3p. Overexpression of circDIDO1 or SOSC2 antagonized the oncogenic role of miR-1307-3p. RGD-Exo-circDIDO1 could efficiently deliver circDIDO1 to increase SOCS2 expression in GC cells. Compared with PBS and RGD-Exo-vector treatment, RGD-Exo-circDIDO1 treatment significantly inhibited the proliferation, migration and invasion of GC cells while promoted cell apoptosis. The therapeutic efficacy of RGD-Exo-circDIDO1 was further confirmed in a mouse xenograft tumor model. In addition, major tissues including the heart, liver, spleen, lungs and kidneys showed no obvious histopathological abnormalities or lesions in the RGD-Exo-circDIDO1 treated group.

**Conclusion:**

Our findings revealed that circDIDO1 suppressed the progression of GC via modulating the miR-1307-3p/SOSC2 axis. Systemic administration of RGD modified, circDIDO1 loaded exosomes repressed the tumorigenicity and aggressiveness of GC both in vitro and in vivo, suggesting that RGD-Exo-circDIDO1 could be used as a feasible nanomedicine for GC therapy.

**Supplementary Information:**

The online version contains supplementary material available at 10.1186/s12967-022-03527-z.

## Introduction

Gastric cancer (GC) is one of the most common malignancies with high morbidity and mortality worldwide and ranks fourth in terms of cancer related deaths [1]. The risk factors for GC include helicobacter pylori infection, age, obesity, excessive intake of salt and nitrates and diets with low fruit and vegetables [2]. Moreover, mutations, differential gene expression, chromosomal aberrations, epigenetic alterations and abnormal intracellular signaling pathways could also induce the development and metastasis of GC [3]. Although continual improvements on clinical diagnosis methods and therapeutic strategies have been made in the past decades, most GC patients are still diagnosed at the intermediate or terminal stage because of the lack of early specific symptoms [2, 4]. Therefore, it is extremely urgent to explore the molecular mechanism of GC progression and find precise biomarkers which could be engineered as therapeutic targets.

Circular RNAs (circRNAs) are an emerging category of non-coding RNA with tissue-specific expression patterns in eukaryotic cells [5]. Compared with traditional linear RNAs, they have high stability due to the covalently closed structure without a 5′cap or a 3′poly A tail [5, 6]. Recently, increasing evidence indicates that circRNAs could participate in cancer development due to their indispensable roles in gene regulation [7, 8], including GC [9], intrahepatic cholangiocarcinoma (ICC) [10], colorectal carcinoma (CRC) [11], breast cancer [12], glioblastoma [13], and others. Numerous studies have explored the molecular mechanisms of circRNAs in cancer progression, showing that circRNAs could function as sponges of microRNAs (miRNAs) [14, 15], interact with RNA-binding proteins [16], mediate gene transcription [17], and encode proteins [18]. Especially, the regulatory network between circRNAs and miRNAs, which is known to function as competing endogenous RNA (ceRNA) [19, 20], has been widely reported in many cancers.

Exosomes, extracellular membrane-derived vesicles with an approximate diameter of 30–200 nm, can be released into the extracellular environment when multivesicular bodies (MVB) fuse with the plasma membrane [21, 22]. In the past decades, many studies have reported that exosomes play a critical role in diverse physiological processes through the intercellular exchange of biomolecules such as proteins, lipids, mRNAs, miRNAs and other non-coding RNAs [23–27]. Meanwhile, because of several unique characteristics such as immune compatibility, low toxicity, nano-scale size, and stability in blood, exosomes can also be engineered to act as a nanocarrier for the delivery of target drugs and nucleic acids. [24]. Notably, exosome-based RNA delivery has shown great promise in the field of cancer therapy. For example, MSCs-Exo can efficiently deliver inhibitors to reduce miR-142-3p levels in vitro and in vivo, which consequently results in a significant inhibitory effect on breast tumor development [25]. Systematic administration of an anti-cancer drug 5-FU and miR-21 inhibitor oligonucleotide (miR-21i) loaded exosomes inhibits colon cancer cell proliferation and reverses drug resistance in 5-FU-resistant colon cancer cells [26].

In our previous study, we have identified a new circRNA, circDIDO1, which is down-regulated in GC tissues. It inhibits GC progression via encoding a DIDO1-529aa protein as PARP1 inhibitor and promoting the RBX1-mediated ubiquitination and degradation of PRDX2 [28]. Considering that the potent tumor suppressor role of circDIDO1, we want to explore the other mechanism responsible for its role and its therapeutic potential. In this study, we reported that circDIDO1 could act as a sponge of miR-1307-3p to regulate SOCS2 expression. Meanwhile, we demonstrated that systematic administration of circDIDO1 by exosome-mediated gene delivery achieved promising anti-cancer effects in vivo and in vitro.

## Material and methods

### Patients and tissue samples

A total of 17 paired tumor tissues and adjacent nontumor tissues (5 cm away from the tumor edge) were collected from the Department of General Surgery, the Affiliated People’s Hospital of Jiangsu University. All the tumor tissues were frozen in liquid nitrogen and stored at −80 °C for further use. Patients in this study had no previous radiotherapy or chemotherapy experience.

### Cell culture and transfection

Human GC cell lines (MGC-803 and HGC-27) were purchased from the Cell Bank of the Chinese Academy of Sciences (Shanghai, China) and Saiku Biological Technology (Guangzhou, China).

All the cells were cultured in RPMI 1640 medium with 10% fetal bovine serum (Bioind, Israel). The HEK-293 T cell line was purchased from the Cell Bank of the Chinese Academy of Sciences (Shanghai, China) and was cultured in high glucose-DMEM medium containing 10% FBS. Cells were incubated at 37 °C with 5% CO_2_ atmosphere. CircDIDO1 and SOCS2 plasmids, NC and miR-1307-3p mimics were transfected into GC cells through Lipofectamine 2000 (Invitrogen, USA). The experiment was conducted according to the manufacturer’s instructions.

### RNA extraction, reverse transcription, and qRT-PCR analysis

Total RNA was extracted from tissues or cultured cells using Trizol reagent (Invitrogen, USA) according to the manufacturer’s protocol. The purity and concentration of total RNA were evaluated using NanoDrop One spectrophotometer (Thermo, USA). RNA was reversely transcribed to cDNA by using the HiScript 1st Strand cDNA Synthesis Kit (Thermo, USA). Subsequent detection of RNA levels was performed with UltraSYBR Mixture kit (Vazyme, China) through Real-time PCR Detection System (CFX96, Bio-Rad, USA). For miRNA expression assay, miRNA primers were synthesized by QIAGEN and total RNA was reversely transcribed using the miScript II Reverse Transcription Kit (QIAGEN, Germany). qRT-PCR amplification for miRNA was performed by using miScript SYBR Green PCR Kit (QIAGEN, Germany). U6 were used as the internal control genes. The relative RNA expression level was calculated using the 2^−△△Ct^ method.

### Isolation and characterization of exosomes

Exosomes were isolated from cell supernatants following our previous protocol [29]. The protein concentration of the exosomes was determined by a BCA protein assay kit (Gibco, China). Size distribution of exosomes was identified through Nanoparticle tracking analysis (NTA) (Nanosight LM10, Particle Metrix, Germany). The morphology of isolated exosomes was observed by using transmission electron microscopy (Philips, Netherlands). The exosomal markers CD9, CD63, TSG101 and the negative control Calnexin were determined by western blot.

### Preparation of RGD-engineered exosomes

293 T cells were transfected with vector and circDIDO1 for 24 h and then cultured in DMEM medium containing 10% exosome-free FBS. After 48 h, cell culture supernatant was collected and exosomes were purified as previously described [29]. Exosomes from the vector (Exo-vector) and DIDO1 overexpression (Exo-circDIDO1) groups were incubated with 50 µg of DSPE-PEG-RGD (Ruixi Biotechnology, China) at 37 ℃ for 30 min to form RGD-Exo-vector and RGD-Exo-circDIDO1, respectively [30].

### RGD-engineered exosomes labeling and internalization

Diluted exosomes (1 mL) were fluorescently labeled using 5 μL of the membrane dye DiL (red, Invitrogen, USA) for 30 min at 37 °C. A total of 1 × 10^4^ cells was cultured on cover glass in 12-well plates. When 60–70% confluence was reached, DiL-labeled exosomes were added and cells were incubated for 12 h. Then cells were washed with PBS and fixed in 4% paraformaldehyde for 20 min at room temperature. Finally, Hoechst 33342 (Sigma, USA) was used for nuclear staining and then intracellular uptake of DiL-labeled exosomes was obtained using a confocal microscope (Beckman Coulter, USA).

### Cell counting and colony formation assays

The transfected cells were seeded in 24-well plates (1 × 10^4^/well) and counted every 24 h for 5 days. For colony formation assay, a total of 1 × 10^3^ cells were seeded into 6-well plates and the medium was replaced every 2 days. After 8 days of incubation, the colonies were fixed with 4% paraformaldehyde and stained with crystal violet. The number of colonies was observed and counted with a microscope.

### Transwell migration and matrigel invasion assays

The cells were harvested and resuspended in serum-free RPMI-1640 medium. Cells (2 × 10^4^ for migration assay and 1 × 10^4^ for invasion assay) in 200 μL of serum-free medium were plated into the upper chambers of a 24-well chamber with an 8.0 μm pore (Corning, USA). For invasion assay, the transwell upper chambers were pre-coated with diluted cold Matrigel (BD Biosciences, USA). The cells were incubated with 600 μL of medium containing 10% FBS in the lower chambers for 24 h (for the migration assay) or 48 h (for the invasion assay). After incubation, cells on the upper surface of the polycarbonate membrane were fixed with 4% paraformaldehyde for 20 min and stained with 0.1% crystal violet for 20 min. The cells were imaged and counted under a microscope.

### RNA immunoprecipitation

RNA immunoprecipitation experiments were performed by the Magna RIP RNA-Binding Protein Immunoprecipitation Kit (Millipore, USA), following the manufacturer’s instructions. Briefly, the transfected cells were pelleted and resuspended in lysis buffer containing a protease inhibitor cocktail and RNase inhibitor. The lysates were immunoprecipitated with magnetic beads conjugated with anti-Ago2 antibody or anti-mouse IgG at 4 °C overnight. Then, the beads were washed by RIP wash buffer and incubated with proteinase K to remove proteins. Finally, the immunoprecipitated RNA was purified and used for qRT-PCR analysis.

### Luciferase reporter assay

The wild-type (WT) or mutant (MUT) luciferase reporter vectors were constructed by Hanheng Biological Technology (Shanghai, China). GC cells were co-transfected with WT or MUT reporter vectors and miRNA mimics by using Lipofectamine 2000 (Invitrogen, USA). After 48 h incubation, cells were harvested, and the luciferase activity was assessed using the Dual-Luciferase Reporter Assay System (Promega, USA).

### Cell apoptosis assay

Cell apoptosis was analyzed by using the Annexin V-Alexa Fluor 647/propidium iodide (PI) apoptosis detection kit (Fcmacs, China). After treatment with RGD-engineered exosomes for 24 h, the cells were detached and dissociated with collagenase. Cell pellets were resuspended in binding buffer and stained with Annexin V-Alexa Fluor 647 and PI for 15 min at room temperature. Cell apoptosis ratio was analyzed by CytoFLEX flow-cytometry (Beckman, USA).

### In vivo animal studies

For the therapeutic study, 15 nude mice were inoculated subcutaneously with MGC-803 cells. After 2 weeks, the mice were randomly divided into 3 groups. PBS, RGD-Exo-vector and RGD-Exo-circDIDO1 were intravenously injected to mice via the tail vein every 4 days for 28 days (30 μg/μL in protein concentration; 200 μL per mouse). The protocol was approved by the Animal Use and Care Committee of Jiangsu University.

### Statistical analysis

GraphPad Prism 7.0 (GraphPad Software, USA) were used for statistical analysis. All data were presented as mean ± standard deviation (SD). The significant difference between different groups was analyzed by Student’s *t*-test and one-way ANOVA test according to actual conditions. *P* < 0.05 was considered as statistically significant.

## Results

### CircDIDO1 is a direct target of miR-1307-3p in GC cells

**Fig. 1:**
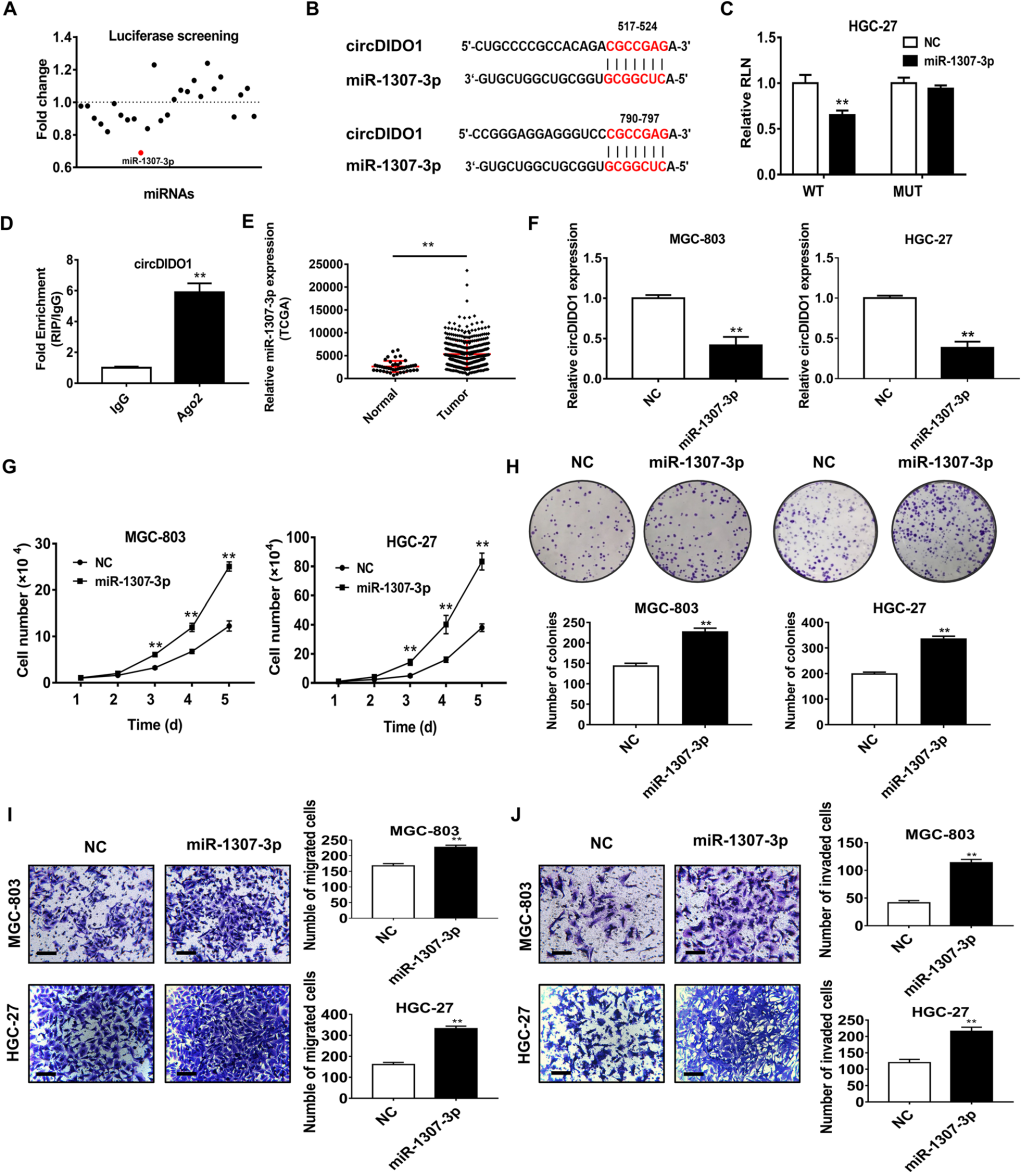
MiR-1307-3p plays oncogenic roles in GC progression. **A** Luciferase reporter assays for screening of miRNAs that potentially regulate circDIDO1. **B** Prediction of binding sites in circDIDO1 for miR-1307-3p. **C** HGC-27 cells were co-transfected with miR-1307-3p and wild-type (WT) or mutant (MUT) luciferase reporter vector for circDIDO1. The luciferase activity was determined as indicated. **D** RNA immunoprecipitation (RIP) assay for the binding of circDIDO1 to Ago2 protein. **E** Analysis of miR-1307-3p expression in tumor tissues and adjacent normal tissues from GC patients by using TCGA data. **F** qRT-PCR analyses of circDIDO1 expression in control and miR-1307-3p overexpressing GC cells. **G**–**J** Cell growth curve, colony formation, transwell migration, and matrigel invasion assays for control and miR-1307-3p overexpressing GC cells. Data are shown as mean ± SD (n = 3, **P < 0.01, *P < 0.05). Scale bars = 100 μm

**Fig. 2:**
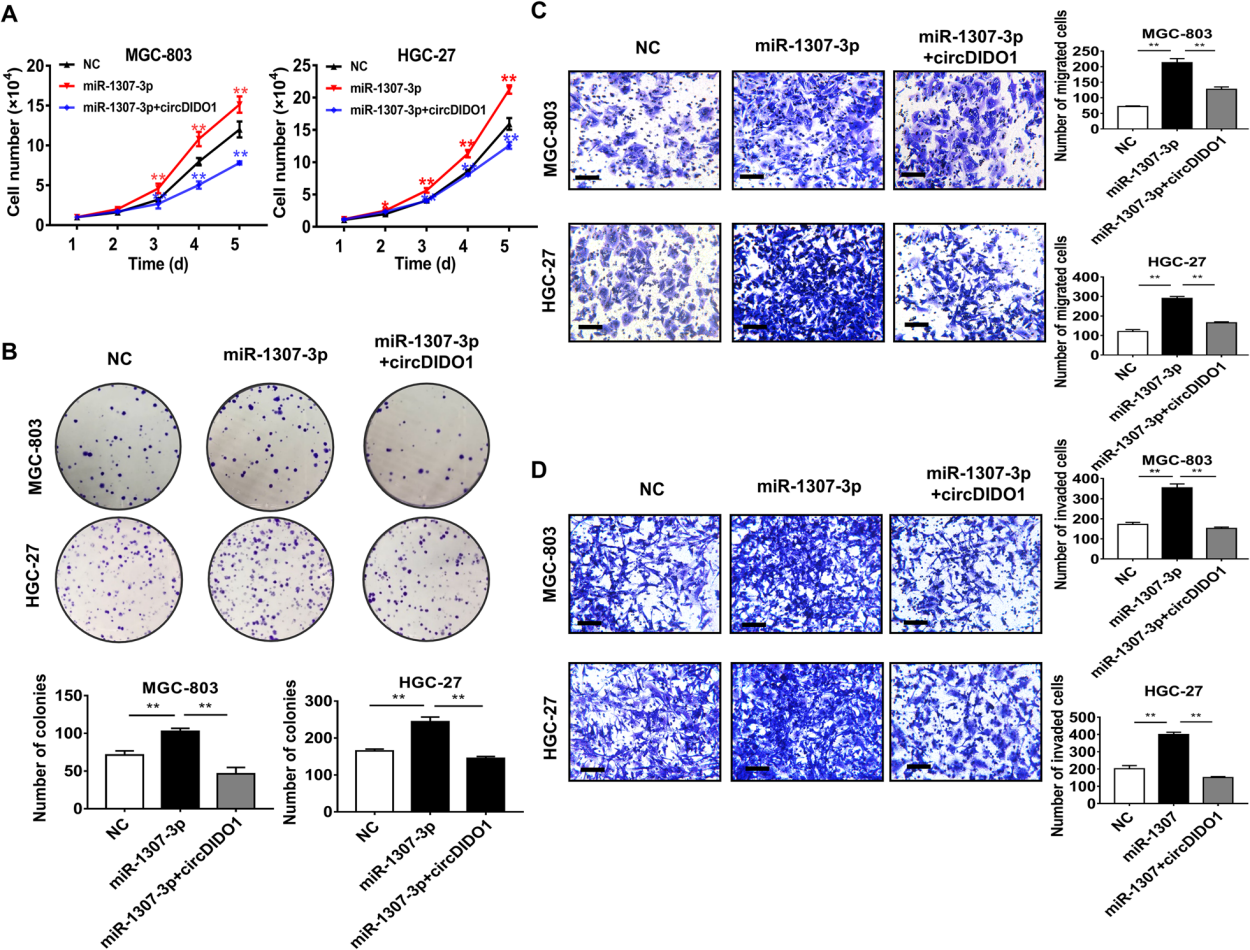
CircDIDO1 acts as a ceRNA for miR-1307-3p. **A**–**D** Cell growth curve, colony formation, transwell migration, and matrigel invasion assays for control, miR-1307-3p, and miR-1307-3p+circDIDO1 transfected GC cells. Data are shown as mean ± SD (n = 3, **P < 0.01, *P < 0.05). Scale bars = 100 μm

To understand the mechanism for circDIDO1 in GC, we used CircInteractome software (https://circinteractome.nia.nih.gov/) to predict the interaction of circDIDO1 with different miRNAs. We found that multiple miRNAs could be potentially sponged by circDIDO1 (Fig. 1A). Among these miRNA candidates, we focused on miR-1307-3p as our luciferase reporter assay results showed a most significant decrease in luciferase activities after co-transfection of miR-1307-3p with the wild-type luciferase reporter vector for circDIDO1. In contrast, miR-1307-3p had no effect on the luciferase activity of the mutant circDIDO1 luciferase reporter vector (Fig. 1B, C). RNA immunoprecipitation was performed to observe whether circDIDO1 binds to miR-1307-3p. The RIP results showed that circDIDO1 could be immunoprecipitated by the antibody against Ago2, a core component of the RNA-induced silencing complex (Fig. 1D). Gene expression analysis by using TCGA data showed that miR-1307-3p was highly expressed in GC tissues compared to adjacent normal tissues (Fig. 1E). MiR-1307-3p has been confirmed to play a critical role in a variety of cancers. To determine whether circDIDO1 regulates GC cells through miR-1307-3p, we conducted a gain-of-function study and found that overexpression of miR-1307-3p significantly inhibited circDIDO1 expression in GC cells, suggesting that circDIDO1 is targeted by miR-1307-3p (Fig. 1F). In addition, miR-1307-3p expression significantly promoted GC cell proliferation, migration, and invasion (Fig. 1G–J). Rescue experiments were performed to confirm the biological effects of circDIDO1/miR-1307-3p axis. The co-transfection of circDIDO1 repressed the promoting role of miR-1307-3p in GC cell proliferation, migration, and invasion (Fig. 2A–D). In summary, these data suggests that circDIDO1 directly binds to miR-1307-3p and serves as a negative regulator of miR-1307-3p.

### MiR-1307-3p regulates the expression of SOSC2

**Fig. 3:**
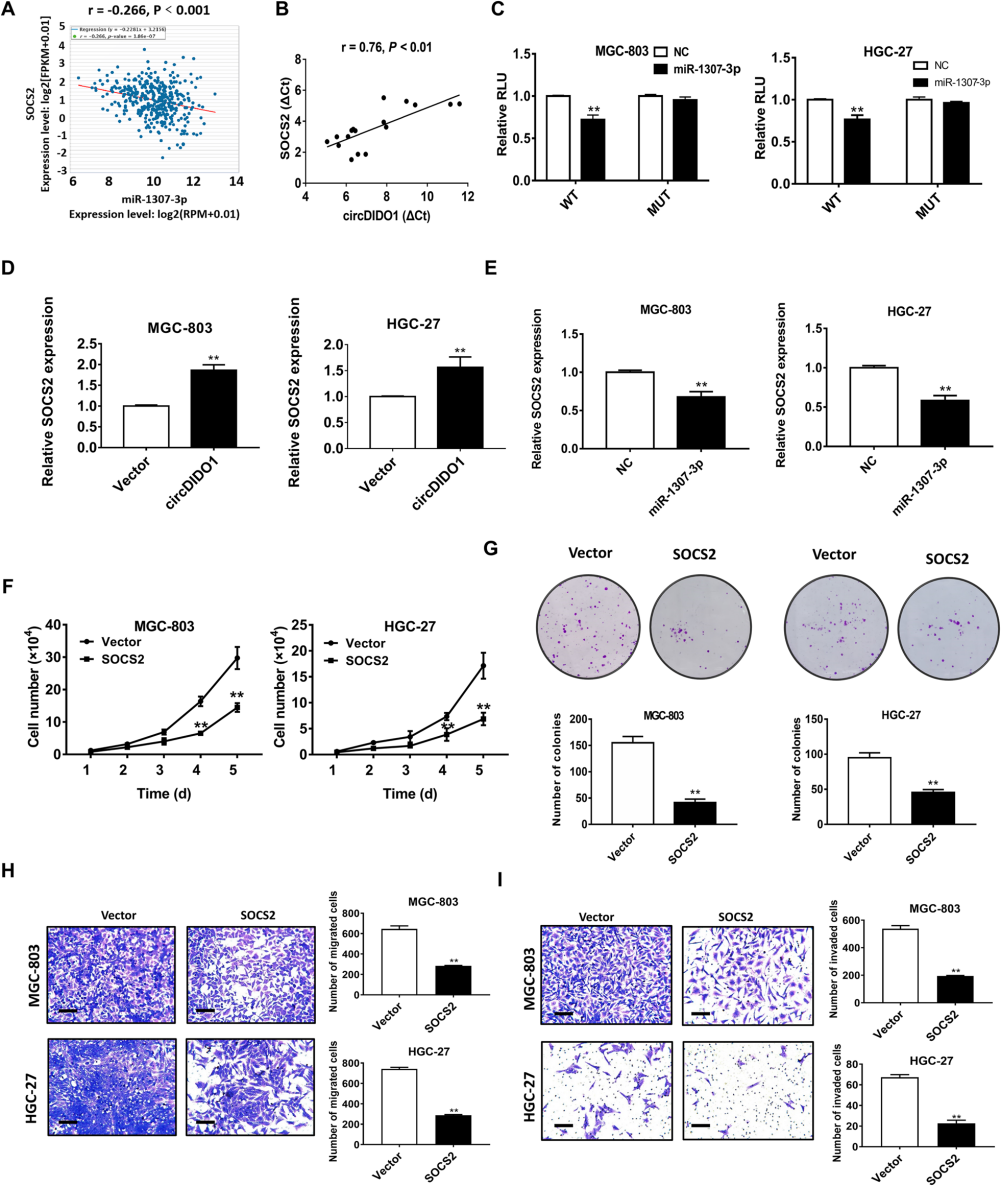
SOCS2 is a target of miR-1307-3p. **A** ENCORI Pan-Cancer Analysis Platform was used to analyze the correlation of miR-1307-3p and SOCS2 in 372 cases of STAD patients. **B** qRT-PCR analyses of the expression of SOCS2 and circDIDO1 in tumor tissues of 17 GC patients. **C** Luciferase reporter assay in GC cells co-transfected with wide-type (WT) or mutant (MUT) SOCS2 plasmid together with miR-1307-3p mimic or NC. **D** The relative level of SOCS2 expression after transfection with circDIDO1 in GC cells. **E** The relative level of SOCS2 expression in control and miR-1307-3p overexpressing GC cells. **F**–**I** Cell growth curve, colony formation, transwell migration, and matrigel invasion assays for control and SOCS2 overexpressing GC cells. Data are shown as mean ± SD (n = 3, **P < 0.01, *P < 0.05). Scale bars = 100 μm

As previously described, the RNA sequencing for control and circDIDO1 overexpressing GC cells and the cluster and pathway analyses for gene expression profiles were performed [28]. We found that 134 genes were up-regulated and 52 genes were down-regulated in circDIDO1 overexpressing group compared to the control group. Suppressor of cytokine signaling 2 (SOCS2) was one of the significantly up-regulated genes. The altered expression of SOCS2 in the circDIDO1 overexpressing group was verified by qRT-PCR [28]. To further seek for the downstream regulatory axis of circDIDO1/miR-1307-3p in GC cells, we also used TargetScan to predict the potential target genes of miR-1307-3p. Combined with RNA sequencing analysis, SOCS2 was confirmed to be a direct target of miR-1307-3p in GC cells. StarBase assay revealed that the expression levels of miR-1307-3p and SOCS2 displayed a significant negative correlation (Fig. 3A). In addition, we performed a correlation analysis between circDIDO1 and SOCS2 in 17 paired GC tissues. The expression of SOCS2 was positively correlated with that of circDIDO1 in GC tissues (Fig. 3B). The co-transfection of miR-1307-3p with the wild-type luciferase reporter vector for SOCS2 significantly decreased the luciferase reporter activity in GC cells. However, these effects were abolished by a mutant SOCS2 luciferase reporter vector (Fig. 3C). Subsequently, qRT-PCR results indicated that circDIDO1 overexpression increased the expression level of SOCS2 (Fig. 3D). In contrast, overexpression of miR-1307-3p significantly inhibited SOCS2 expression in GC cells (Fig. 3E). These findings suggest that circDIDO1 sponges miR-1307-3p to upregulate SOCS2 expression. Moreover, the results of gain-of-function studies revealed that SOCS2 overexpression inhibited GC cell proliferation, migration, and invasion (Fig. 3F–I).

### CircDIDO1 represses gastric cancer progression through regulating the miR-1307-3p/SOCS2 axis

**Fig. 4:**
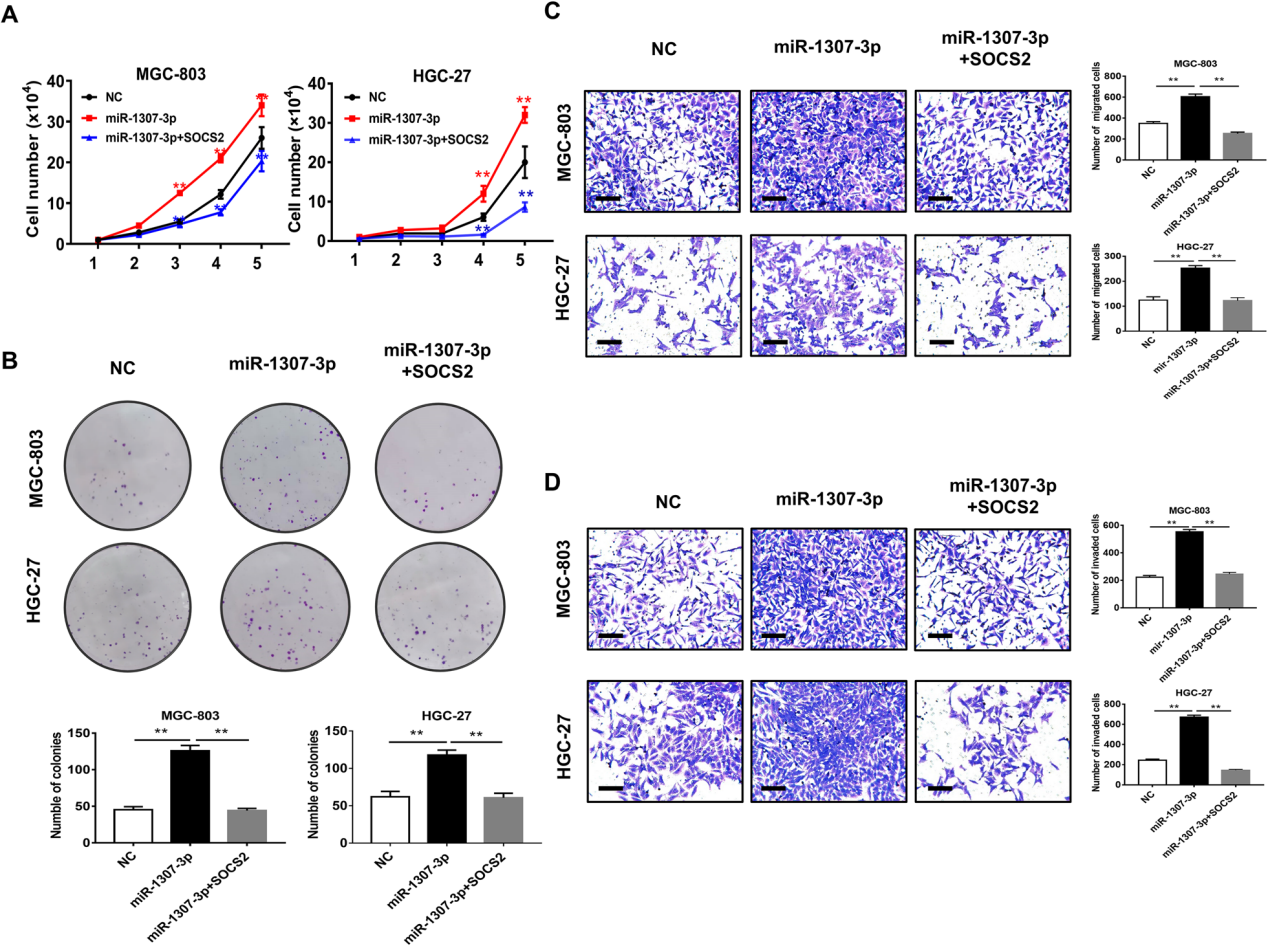
SOCS2 reverses the oncogenic roles of miR-1307-3p in GC cells. **A**–**D** Cell growth curve, colony formation, transwell migration, and matrigel invasion assays for control, miR-1307-3p, and miR-1307-3p+SOCS2 transfected GC cells. Data are shown as mean ± SD (n = 3, **P < 0.01, *P < 0.05). Scale bars = 100 μm

We further performed rescue studies to explore whether circDIDO1 regulates gastric cancer progression via the miR-1307-3p/SOCS2 axis and the results of rescue experiments showed that SOCS2 overexpression partially reversed the promoting roles of miR-1307-3p in GC cell proliferation, migration, and invasion (Fig. 4A–D). Collectively, our findings provided the first evidence that circDIDO1 regulates SOCS2 expression through miR-1307-3p, thereby inhibiting GC progression.

### Isolation and characterization of RGD-Exo-circDIDO1

**Fig. 5:**
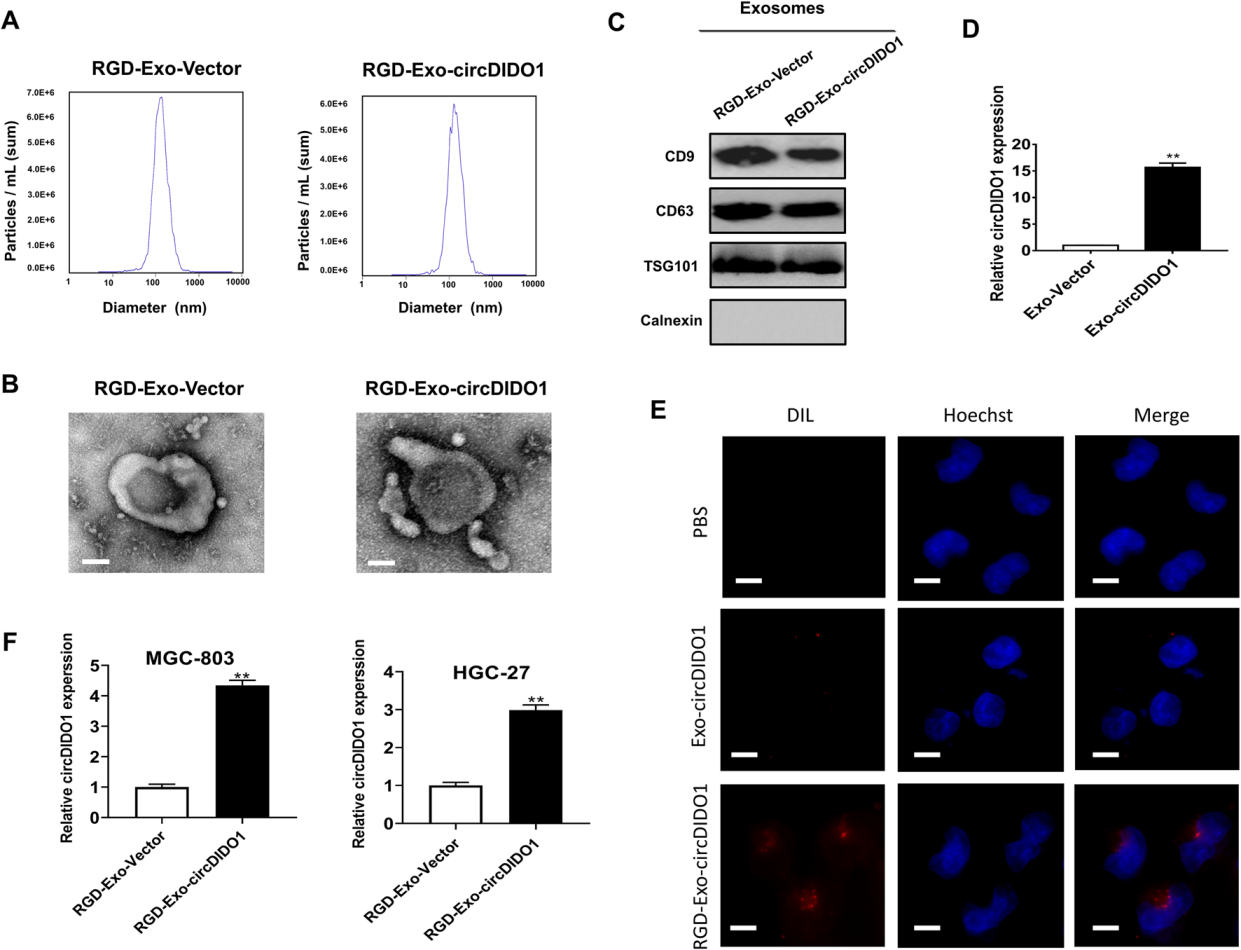
Characterization and the targeting ability of RGD-Exo-circDIDO1. **A** The size distribution of RGD-Exo-circDIDO1 was detected by nanosight tracking analysis. **B** The morphology of exosomes was observed using a transmission electron microscope (scale bar = 40 nm). **C** The exosomal markers were analyzed by western blotting. **D** qRT-PCR analyses of circDIDO1 expression in RGD-Exo-circDIDO1. **E** The internalization of DiI-labeled RGD-Exo-circDIDO1 in GC cells was observed by confocal fluorescence microscope (scale bar = 20 μm). **F** qRT-PCR analyses of circDIDO1 expression in RGD-Exo-circDIDO1 treated GC cells. Data are shown as mean ± SD (n = 3, **P < 0.01, *P < 0.05)

The potent suppressive role of circDIDO1 in GC progression led us to hypothesize that it may be a potential therapeutic target for GC. To this end, we constructed circDIDO1-loaded, RGD-modified exosomes (RGD-Exo-circDIDO1) and investigated its effect on GC therapy. RGD-Exo-circDIDO1 were extracted and purified through differential centrifugation from the cell culture supernatant, which were subsequently characterized by nanosight tracking analysis, transmission electron microscopy and western blot. The diameter of most RGD-Exo-circDIDO1 ranged from 100 to 130 nm, and the average size was found to be approximately 120 nm (Fig. 5A). We found that the exosome exhibited a sphere-shaped morphology (Fig. 5B). Exosomal markers such as CD9, CD63 and TSG101 were abundant in the RGD-Exo-circDIDO1 while the negative control Calnexin was not observed in the RGD-Exo-circDIDO1 (Fig. 5C). Subsequently, we extracted the total RNA of the exosomes and demonstrated by qRT-PCR that the circDIDO1 expression level was significantly increased in Exo-circDIDO1 (Fig. 5D). To evaluate the cellular tropism of RGD-Exo-circDIDO1 in vitro, RGD-Exo-circDIDO1 was labeled with DiL and co-cultured with GC cells for 12 h. Confocal microscope results showed that the internalization of Exo-circDIDO1 modified with RGD into GC cells was more efficient compared to Exo-circDIDO1 without RGD modification (Fig. 5E). RGD-Exo-circDIDO1 had the efficient delivery ability of circDIDO1, which is indicated by the significant increase of circDIDO1 level in GC cells treated with RGD-Exo-circDIDO1 compared to the control exosome group (Fig. 5F). Overall, the RGD-modified exosomes with a high level of circDIDO1 could be successfully prepared for further study.

### Anti-tumor effect of RGD-Exo-circDIDO1 in vitro

**Fig. 6:**
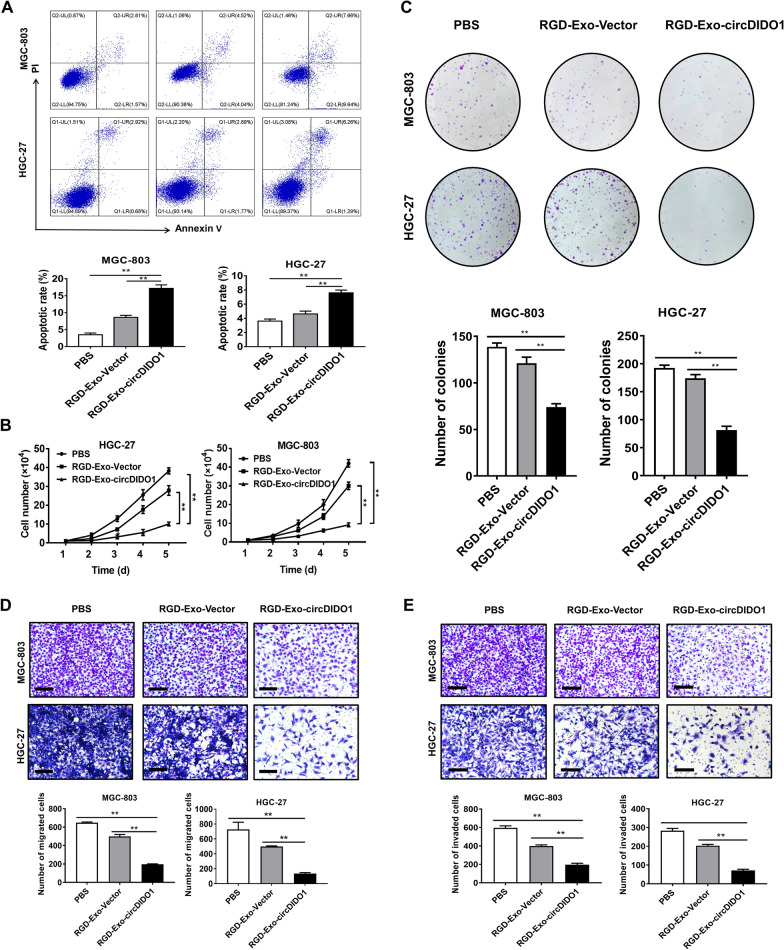
RGD-Exo-circDIDO1 treatment inhibits GC cell growth while promotes apoptosis. **A**–**E** Flow cytometry analysis of cell apoptosis (**A**), cell growth curve (**B**), colony formation (**C**), transwell migration (**D**) and matrigel invasion assays (**E**) were performed for PBS, RGD-Exo, and RGD-Exo-circDIDO1 treated GC cells. Data are shown as mean ± SD (n = 3, **P < 0.01, *P < 0.05). Scale bars = 100 μm

**Fig. 7:**
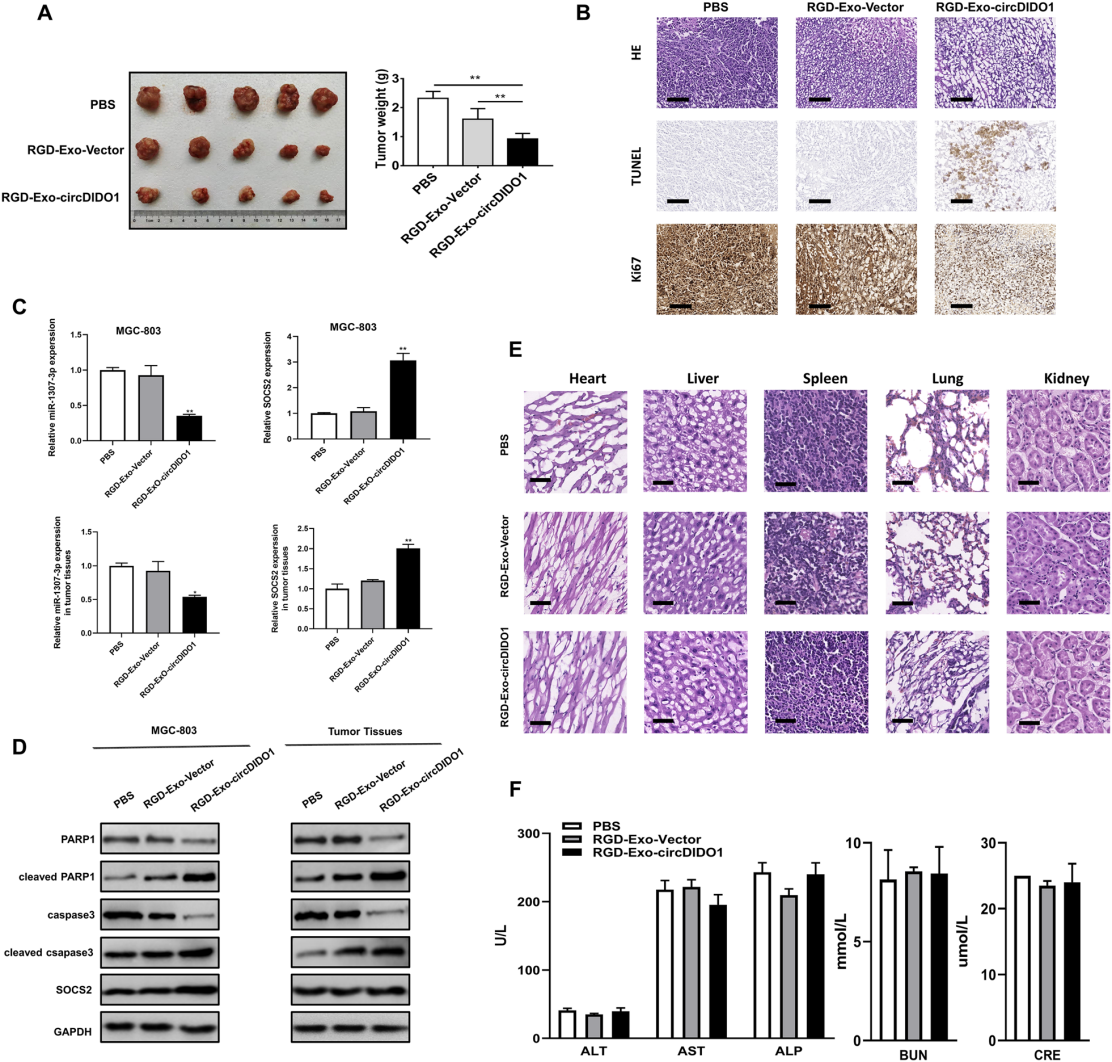
RGD-Exo-circDIDO1 efficiently inhibits GC growth in mice. **A** PBS, RGD-Exo, and RGD-Exo-circDIDO1 were intravenously injected into nude mice bearing subcutaneous xenograft tumors. At 28 days after treatment, the mice were sacrificed and the volume and weight of tumors were recorded. Data are shown as mean ± SD (n = 5 for each group, **P < 0.01). **B** HE, Ki-67 and TUNEL staining of xenograft tumor tissues treated with PBS, RGD-Exo, and RGD-Exo-circDIDO1. Scale bars = 100 μm. **C** qRT-PCR assays were used to examine miR-1307-3p and SOCS2 expression in GC cells and tissues treated with PBS, RGD-Exo, and RGD-Exo-circDIDO1. Data are shown as mean ± SD (n = 3, **P < 0.01, *P < 0.05). **D** Western blotting analyses of treated GC cells and tumor tissues. **E** Histopathological analyses of heart, liver, spleen, lung and kidney sections stained with HE. Scale bars = 60 μm. **F** Blood biochemistry for liver function markers: ALT, AST and ALP and kidney function markers: BUN and CRE. Data are shown as mean ± SD (n = 5 for each group)

To further investigate the functional role of RGD-Exo-circDIDO1 in GC cells, the ability of PBS, RGD-Exo-vector and RGD-Exo-circDIDO1 in GC cell apoptosis after 12 h treatment was assessed. The annexin V/PI staining and flow cytometry results indicated a significant increase of apoptotic GC cells in the RGD-Exo-circDIDO1 treatment group compared with the other two groups (Fig. 6A). The RGD-Exo showed minimal therapy effect, which indicated that the anti-tumor effect was from circDIDO1, instead of RGD-Exo. In addition, compared with PBS and RGD-Exo-vector treatment, RGD-Exo-circDIDO1 treatment significantly inhibited the proliferation, migration and invasion of GC cells (Fig. 6B–E). Western blot results also revealed that exosome-mediated delivery of circDIDO1 resulted in increased levels of cleaved caspase 3 and cleaved PARP1 in GC cells (Fig. 7D, left panel), both of which are markers for cells undergoing apoptosis. These results suggest that compared with PBS and RGD-Exo-vector treatment, RGD-Exo-circDIDO1 can induce an efficient anti-tumor effect.

### Systemic administration of RGD-Exo-circDIDO1 inhibits GC growth in mice

To investigate the anti-tumor effect of RGD-Exo-circDIDO1 in vivo, we established a xenograft mouse model of gastric cancer by subcutaneously inoculating MGC-803 cells into nude mice. Tumor growth analysis revealed that exosomes loaded with circDIDO1 induced a significant decrease in tumor volume and weight compared to the RGD-Exo-vector and PBS treated mice (Fig. 7A). As shown in Fig. 7B, immunohistochemical staining results revealed the decrease of Ki-67-positive proliferating cells and increase of TUNEL-positive apoptotic cells in the RGD-Exo-circDIDO1 treated group compared to the other two groups. These results showed that RGD-Exo-circDIDO1 treatment remarkably suppressed tumor growth. In summary, the therapeutic efficacy of RGD-Exo-circDIDO1 was confirmed in a mouse xenograft tumor model.

### Engineered exosome-based delivery of circDIDO1 inhibites GC growth through MiR-1307-3p/SOCS2 axis

As shown in Fig. 7, exosome-mediated delivery of circDIDO1 resulted in a significant increase of SOCS2 level compared to the GC cells treated with unloaded-Exo or PBS (Fig. 7C, upper panel). The similar effect was further examined in vivo. The level of miR-1307-3p was downregulated in tumor tissues treated with RGD-Exo-circDIDO1, while SOCS2 was upregulated subsequently (Fig. 7C, lower panel). In accordance, the protein level of SOCS2 was increased by exosome-mediated delivery of circDIDO1 in treated GC cells and tissues. Besides, the expressions of apoptosis-related proteins was measured in tumor tissues by western blot. The results revealed that RGD-Exo-circDIDO1 treatment led to an obvious increase of cleaved caspase 3 and cleaved PARP1 protein levels in tumor tissues (Fig. 7D, right panel). Collectively, these results further confirm that miR-1307-3p/SOCS2 axis is essential for the circDIDO1-mediated tumor suppression effect in GC.

### In vivo safety evaluation for engineered exosome-mediated delivery of circDIDO1 in mice

In addition to treatment efficacy, toxicity is another vital parameter of an excellent delivery vehicle. For safety purpose, we evaluated the systematic toxicity of engineered exosomes in nude mice after intravenous tail vein injection at a dosage of 200 μL per mouse every 4 days for 28 days (30 μg/μL in protein concentration). Compared with PBS treated group, neither death nor a significant difference in body weight was observed in the RGD-Exo-circDIDO1 treated group during the study period (data not shown). Moreover, we further detected the potential pathological lesions induced by engineered exosomes on major organs (i.e., hearts, livers, spleens, lungs, and kidneys). Blood biochemistry and hematology analysis were performed to investigate any potential toxic effect of RGD-Exo-circDIDO1 on the treated mice. On the 28th day after the first injection, all the mice were euthanized, and their major organs and blood were collected for H&E staining, histology analysis, and blood test. As shown in Fig. 7E, major organs had no obvious histopathological abnormalities or lesions among the RGD-Exo-circDIDO1 treated group, which suggested no evidence of inflammatory response caused by RGD-Exo-circDIDO1. Furthermore, no significant hepatic or renal toxicity induced by RGD-Exo-circDIDO1 was observed, as indicated by normal values of liver function markers (ALT, AST, and ALP) and kidney function markers (BUN and CRE) (Fig. 7F). CCK8 assays were performed to assess the potential toxicity of RGD-Exo-circDIDO1 to normal gastric mucosa epithelial cells (GES-1). No toxicity was observed since cell viabilities in all groups remained above 95% at 24 h, 48 h and 96 h after treatment (Additional file 1: Figure S1). All the above results suggest that RGD-Exo-circDIDO1 treatment could be used as a promising approach for GC therapy without significant side effects.

**Fig. 8:**
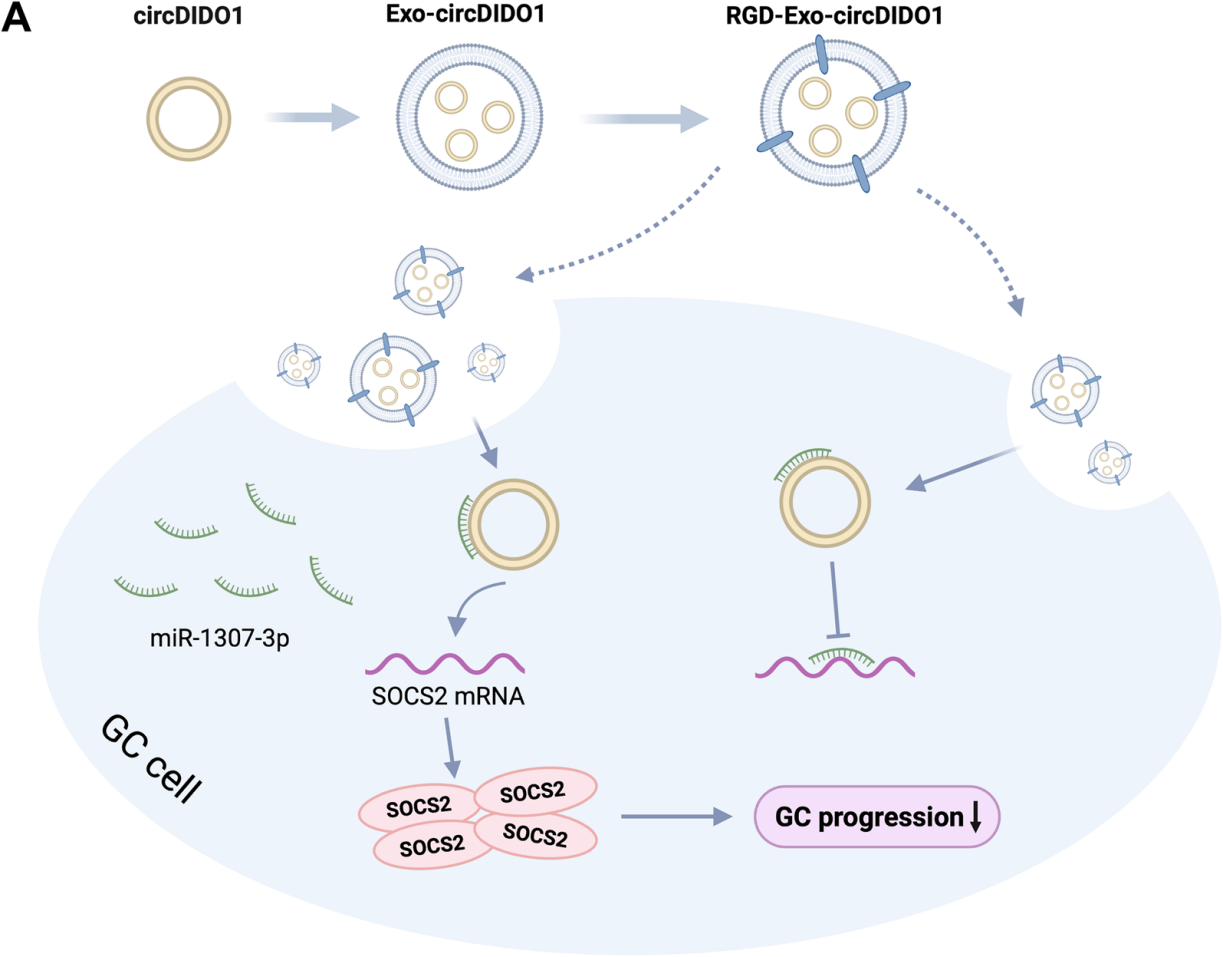
Schematic diagram shows that RGD-Exo-circDIDO1 inhibits GC progression through miR-1307-3p/SOSC2 axis. CircDIDO1 is delivered to GC cells by RGD-modified engineered exosomes. CircDIDO1 sponges miR-1307-3p to upregulate SOCS2 expression, thus inhibiting GC progression

## Discussion

Increasing evidence suggests that circRNAs widely participate in GC development. For instance, circ-RanGAP1 regulates VEGFA expression by targeting miR-877-3p to facilitate GC invasion and metastasis [9]. CircLMO7 acts as a miR-30a-3p sponge to inhibit GC progression by regulating the WNT2/β-Catenin pathway [31]. In our previous study, circDIDO1 was identified as a novel circRNA which was down-regulated in GC [28], but its molecular mechanisms and potential application value in GC were not fully revealed. Our present research verified that circDIDO1 could function as a sponge of miR-1307-3p to induce the expression of SOCS2, thereby suppressing GC progression. Furthermore, we found that exosomes can be used as nanocarriers to deliver circDIDO1 to GC and show an efficient anti-tumor effect (Fig. 8).

Our study has revealed the importance of circDIDO1 in tumor development. Many studies suggest that circRNAs could function as miRNA sponges to modulate GC development [9, 31]. Since the bioinformatic predictions showed that circDIDO1 has the potential to bind to Ago2 protein, we speculated that circDIDO1 might act as a ceRNA to compete for miRNA binding, thereby negatively regulating miRNA activity. Subsequently, the results confirmed that circDIDO1 inhibits GC progression by sponging miR-1307-3p. A few previous studies have shown that miR-1307-3p plays an important role in cancer progression. It has been proved that miR-1307-3p is upregulated in breast cancer tissues and significantly contributes to breast cancer development by targeting SMYD4 [32]. Another study indicates that circPPP1CB inhibits human bladder cancer growth, metastasis, and EMT process by modulating the miR-1307-3p/SMG1 axis [33]. However, the biological role of miR-1307-3p in GC has not been reported yet. Our findings suggest that up-regulation of miR-1307-3p in GC promotes tumor progression and circDIDO1 could reverse the effects of miR-1307-3p, which is consistent with other studies on miR-1307-3p [32, 33].

SOCS2, which is a member of the suppressor of cytokine signaling (SOCS) family, can repress the cytokine-induced signaling transduction, thus inhibiting cancer progression [34, 35]. In addition, SOCS2 has been investigated as the molecular target of diverse miRNAs, including miR-196a/miR-196b [36], miR-194 [37] and miR-767-5p [38]. Herein, we validated that the expression level of SOCS2 was decreased in GC, and similar to circDIDO1, overexpression of SOCS2 suppressed GC cell proliferation, migration and invasion. More importantly, circDIDO1 could positively modulate the expression of SOCS2 in GC cells via acting as a sponge of miR-1307-3p. This ceRNA regulatory network might provide a new therapeutic target for GC treatment.

Targeting circRNAs has been suggested as a new approach for cancer therapy [39–41]. For instance, cell-penetrating inhibitory peptides that block the interaction between oncogenic circRNAs and proteins have been used to inhibit the growth and metastasis of GC cells [41]. Similarly, synthetic circRNA that mimics its natural counterpart has been produced to suppress GC cell proliferation by acting as miRNA sponge [42]. Following this strategy, we further explored the possibility of utilizing circDIDO1 as a target for GC therapy. In recent years, exosomes have emerged as a new vehicle for drug delivery [43]. Exosome-mediated deliveries of siRNA, miRNAs or anti-miRNA oligos, and drugs, have been widely tested in the treatment of various cancers. RGD modification further improves the targeting ability and therapeutic effect of exosome-mediated drug delivery [44, 45]. Thus, we prepared RGD modified, circDIDO1 loaded exosomes by using active loading and passive modification strategies. Our findings revealed that systemic administration of RGD modified, circDIDO1 loaded exosomes could efficiently repress the tumorigenicity and aggressiveness of GC cells. More importantly, the engineered exosome-based delivery system could significantly up-regulate SOCS2 expression in treated GC cells and tissues. These results indicate that miR-1307-3p/SOCS2 axis plays an essential role in the anti-tumor effect of exosome-mediated delivery of circDIDO1. Finally, the safety of the engineered exosomes in mice after treatment was evaluated, and in vivo studies demonstrated that no significant cytotoxicity or systemic toxicity was induced by the engineered exosomes.

During the last decade, several clinical trials using engineered exosomes to activate immune response or deliver therapeutic nucleic acids have been conducted [46]. For example, a phase-2 clinical trial verified the activation of NK cells after dendritic cell-derived exosome administration achieved immunotherapeutic effect in advanced non-small cell lung cancer patients without obvious toxicity [47]. In addition, MSC-derived exosomes loaded with KrasG12D siRNA are now being utilized in a phase-1 clinical trial against pancreatic cancer (NCT 03608631) [48]. Therefore, considering that RGD-modified, circDIDO1-loaded exosomes we constructed have shown significant anti-tumor effects without observable toxicity in vitro and in vivo, exosome-based circRNA delivery could be developed as a promising therapeutic strategy with high biosafety for GC.

Due to their stability, safety and homing characteristics, exosomes have been widely explored as delivery vehicles for a variety of cargos [49]. The accumulation of exosomes in the liver after administration provides a venue to load them with antioxidants to reduce endogenous ROS generation [50, 51]. Eftekhari et al*.* have reported an enhanced antioxidative effect after nano-carrier encapsulation to achieve better hepatoprotective effect [52]. Therefore, the application of exosomes to deliver antioxidants may reduce drug-induced hepatotoxicity caused by ROS generation and subsequent NF-κB activation [53, 54]. In addition, identifying effective serum markers is important to improve the survival rate of GC patients. Ma et al*.* demonstrate that circDIDO1 expression level in serum exosomes could be used as a promising indicator for liver failure [55]. In addition, serum miR-1307-3p has been suggested as an effective diagnostic marker for early breast cancer [56]. Considering the critical role of circDIDO1 and miR-1307-3p in GC progression, serum circDIDO1 and miR-1307-3p have great potential as measurable biomarkers for GC screening and early diagnosis. Furthermore, peritoneal metastasis mouse model will be established to further confirm the anti-tumor efficiency of RGD-Exo-circDIDO1 in GC metastasis.

## Conclusion

In summary, our findings discovered the biological role of the circDIDO1/miR-1307-3P/SOCS2 signaling pathway in GC progression. We also successfully developed a strategy that utilized the engineered exosomes to deliver a tumor suppressive circRNA for GC treatment.

## Supplementary Information

**Additional file 1: Figure S1. **The viabilities of normal gastric mucosa epithelial cells treated with PBS, RGD-Exo, and RGD-Exo-circDIDO1 were measured by CCK8 assays. Data are shown as mean ± SD (n=3).

## Data Availability

All data generated or analyzed during this study are included in this published article.

## Abbreviations

GC: Gastric cancer
circRNAs: Circular RNAs
miRNAs: MicroRNAs
ceRNA: Competing endogenous RNA
Exo: Exosome
MVB: Multivesicular bodies
NTA: Nanoparticle tracking analysis
WT: Wild-type
MUT: Mutant
SOCS2: Suppressor of cytokine signaling 2
