# Supplementary figures and images for: Engineered exosome-mediated delivery of circDIDO1 inhibits gastric cancer progression via regulation of MiR-1307-3p/SOCS2 Axis
Source: J Transl Med. 2022 Jul 21;20:326. doi: 10.1186/s12967-022-03527-z (PMC9306104; doi:10.1186/s12967-022-03527-z)

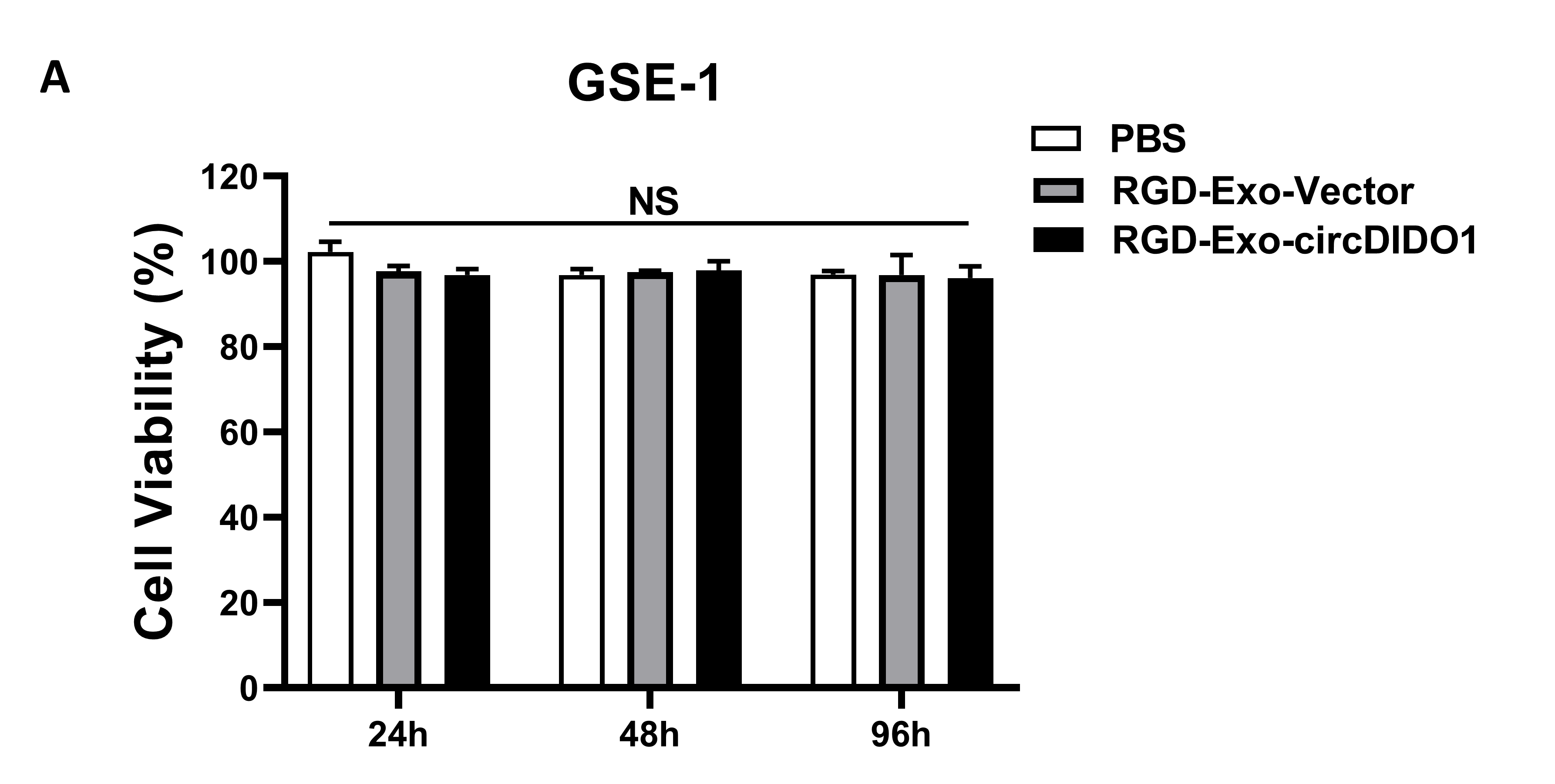

Supplement: Supplementary file 1 — Additional file 1: Figure S1. The viabilities of normal gastric mucosa epithelial cells treated with PBS, RGD-Exo, and RGD-Exo-circDIDO1 were measured by CCK8 assays. Data are shown as mean ± SD (n=3). [file 12967_2022_3527_MOESM1_ESM.tif]
